# Supplementary material for: New Insights into Endogenous Retrovirus-K Transcripts in Amyotrophic Lateral Sclerosis
Source: Int J Mol Sci. 2024 Jan 26;25(3):1549. doi: 10.3390/ijms25031549 (PMC10855536; doi:10.3390/ijms25031549)
Supplement: Supplementary file 1 [file ijms-25-01549-s001.zip › 20240111_Supplementary material.pdf]

Article

New Insights into Endogenous Retrovirus-K Transcripts in Amyotrophic Lateral Sclerosis

Laura Moreno-Martinez [1,2,3,4,†], Sofía Macías-Redondo [5,†,‡], Mark Strunk [5], María Isabel Guillén-Antonini [3], Christian Lunetta [6,7,8], Claudia Tarlarini [8], Silvana Penco [8], Ana Cristina Calvo [1,2,3,4], Rosario Osta [1,2,3,4] and Jon Schoorlemmer [3,5,9,*]

1 Laboratory of Genetics and Biochemistry (LAGENBIO), Faculty of Veterinary, University of Zaragoza, Miguel Servet 177, 50013 Zaragoza, Spain; lauramm@unizar.es (L.M.-M.); osta@unizar.es (R.O.)
2 Centro de Investigación Biomédica en Red en Enfermedades Neurodegenerativas, Instituto de Salud Carlos III (CIBER-CIBERNED-ISCIII), 28029 Madrid, Spain
3 Instituto de Investigación Sanitaria Aragón (IIS Aragón), 50009 Zaragoza, Spain
4 Instituto Agroalimentario de Aragón (IA2), University of Zaragoza-CITA, C/Miguel, Servet 177, 50013 Zaragoza, Spain
5 Instituto Aragonés de Ciencias de la Salud (IACS), Centro de Investigación Biomédica de Aragón (CIBA), 50009 Zaragoza, Spain; smacias@certest.es (S.M.-R.)
6 NEMO (NEuroMuscular Omnicentre) Clinical Center, Fondazione Serena Onlus, 20162 Milan, Italy
7 Neurorehabilitation Department of Milano Institute, Istituti Clinici Scientifici Maugeri IRCCS, 20138 Milan, Italy
8 Medical Genetics Unit, Department of Laboratory Medicine, ASST Grande Ospedale Metropolitano Niguarda, 20162 Milan, Italy; claudia.tarlarini@ospedaleniguarda.it (C.T.)
9 ARAID Foundation, 50009 Zaragoza, Spain
* Correspondence: jonas@araid.es
† These authors contributed equally to this work.
‡ Current address: Certest Biotec, 50840 Zaragoza, Spain.

Supplementary figures

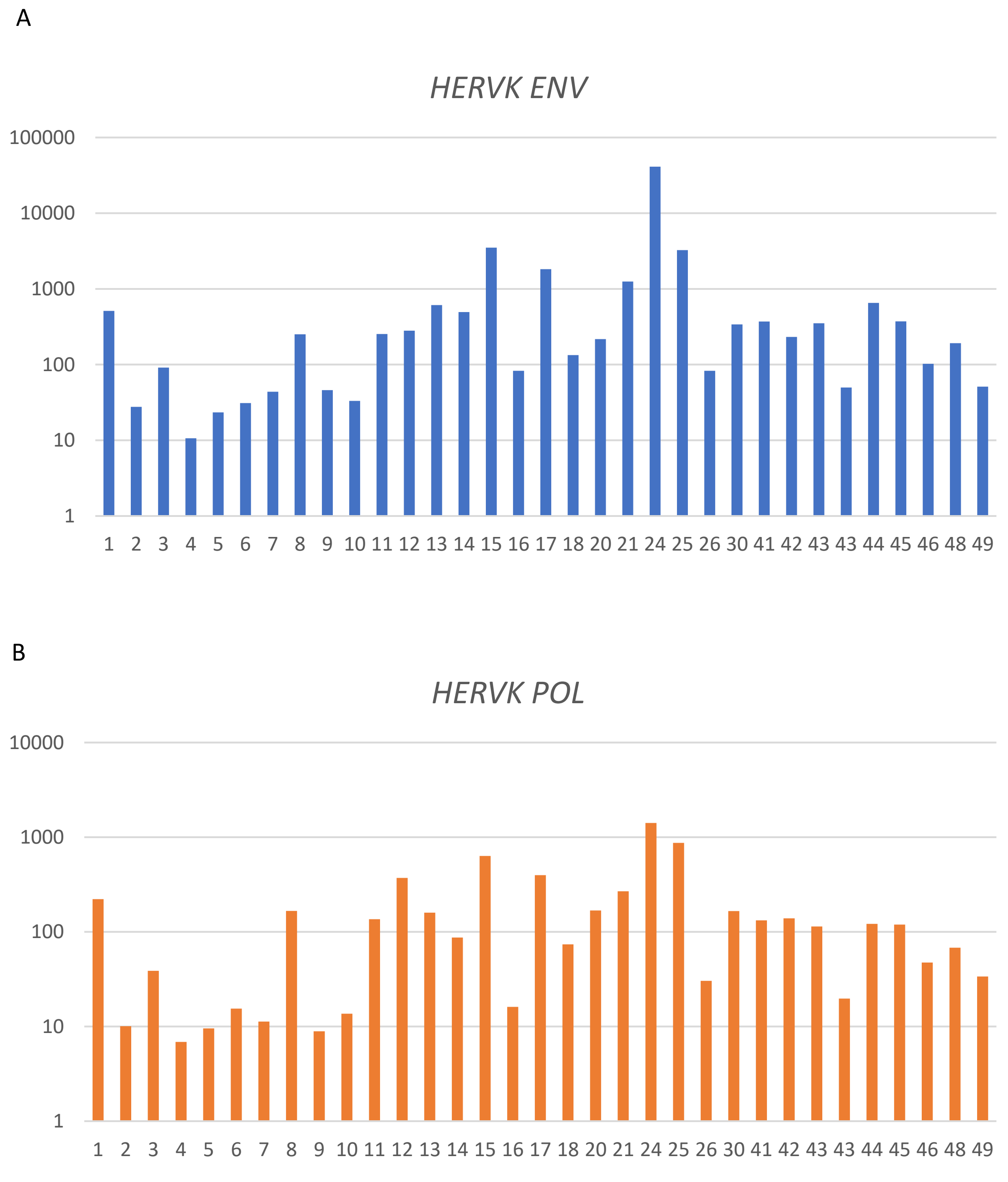

Figure S1. Analysis of amplification as a function of RT activity. RNA samples (n=33) were reverse transcribed with or without enzyme (the latter indicated as mock). Resulting templates were used to amplify either *HERVK ENV* (A) or *POL* (B) genes by qPCR, the results of which were registered as Ct values. The difference between the two conditions was calculated (ΔCt) and recalculated as fold change. The figure shows the fold change calculated between the conditions (+/-) RTase for each sample (X axis) represented on a logarithmic scale (Y axis).

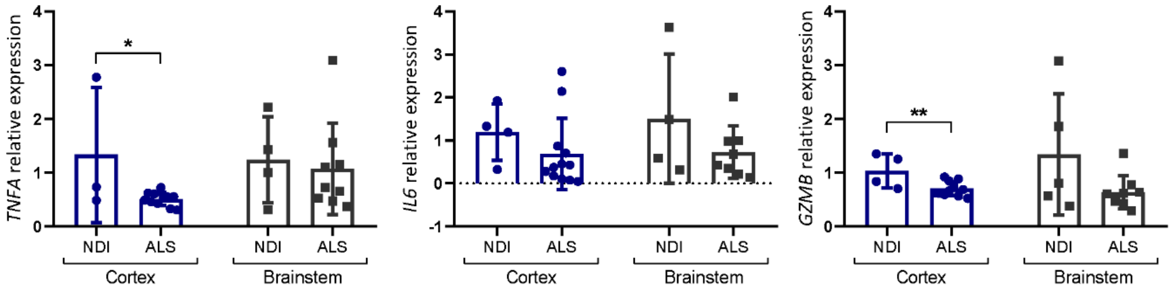

Figure S2. Inflammation-related gene expression in ALS brain. Expression analysis of the genes indicated was carried out by qPCR on cDNA samples prepared from brain samples of ALS patients and controls (NDI). Results were normalized using *GAPDH* and *RPL19* as reference genes, calculated using the $2^{-\Delta\Delta Ct}$ method, and represented as the fold expression compared to the mean expression level in controls. ALS patient samples as well as control (NDI) samples were prepared from either cerebral cortex or brainstem. Cortex ALS n= 10; Cortex NDI n=3; Brainstem ALS n=9; Brainstem NDI n=4. t-student; * $p < 0.05$, ** $p < 0.01$.

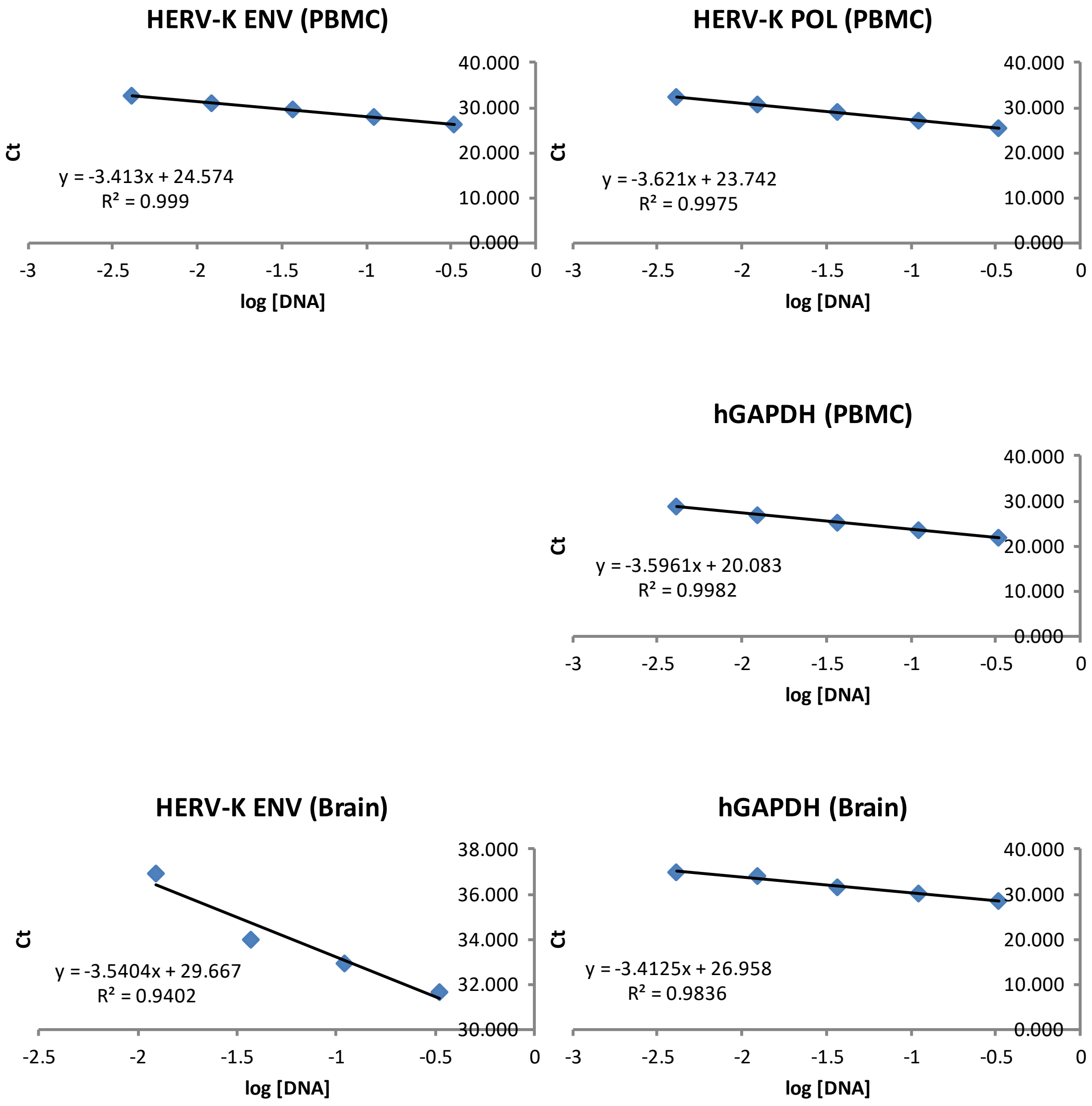

Figure S3. Linear standard curves for *HERVK* qPCR. Expression analysis of Human endogenous retrovirus *ENV*, *POL* genes or human *GAPDH* was carried out as described in the Materials and Methods section and the legend to Figure 1, using the indicated templates: a random PBMC sample, a brain sample with an about average RQI.

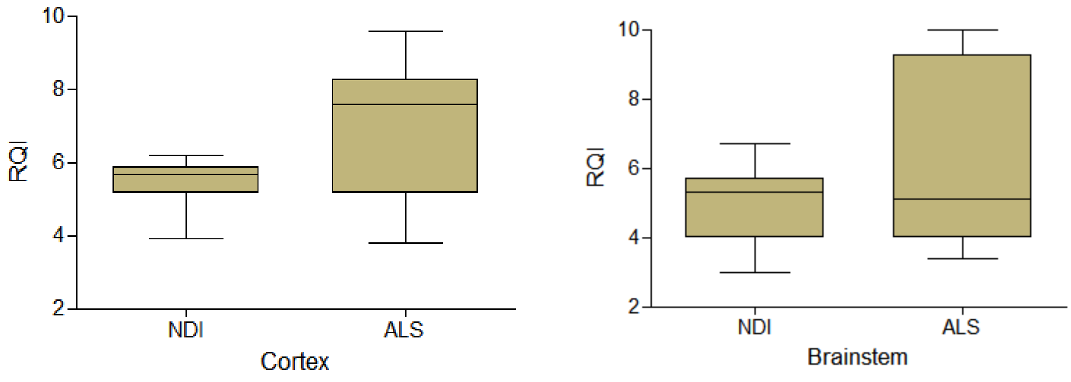

Figure S4. Mean RNA quality indicator (RQI) values for RNA of post-mortem brain samples in the different groups are 6.90 (ALS Cortex), 6.08 (ALS Brainstem), 5.45 (NDI Cortex) and 4.96 (NDI Brainstem). No statistical differences were encountered between ALS patients and NDI groups, p=0.298 and 0.064 (Mann-Whitney U test) for brainstem and cortex, respectively.

Supplementary tables

Table S1. Total reads per individual sample.

	Sample	Total reads
Cortex ALS	1	123803
	2	115687
	3	75918
	4	61477
	5	69297
Medulla ALS	6	102465
	7	106010
	8	95167
	9	123586
	10	89224
Cortex NDI	11	132295
	12	82548
	13	108058
	14	52193
Medulla NDI	15	96001
	16	89805
	17	64168
	18	60450
PBMC NDI	19	43876
	20	136704
	21	76004
	22	75899
	23	52772
	24	62607
	25	112805
	26	106904
	27	54612
	28	61610
PBMC ALS	29	25286
	30	67880
	31	59762
	32	77411
	33	70704
	34	54782
	35	77230
	36	67173
	37	56448
	38	73413

Table S2. Nomenclature and genomic localization of HERVK (HML-2) copies analyzed.

Region indicator used	Nomenclature (Subramanian)	Locus GRch 38.92	Human specific
HERV-K_copy_chr1-1	1p31.1	chr1:75382167-75382531	Yes
HERV-K_copy_chr1-2	1q22	chr1:155627592-155627956	Yes
HERV-K_copy_chr1-3	1q23.3	chr1:160698725-160699089	
HERV-K_copy_chr1-4	1q32.2	chr1:207636028-207636392	
HERV-K_copy_chr2	2q21.1	chr2:129962884-129963248	Yes
HERV-K_copy_chr3-1	3p25.3	chr3:9848603-9848969	
HERV-K_copy_chr3-2	3q12.3	chr3:101699723-101700087	
HERV-K_copy_chr3-3	3q13.2	chr3:113025195-113025559	Yes
HERV-K_copy_chr3-4	3q21.2	chr3:125898474-125898833	Yes
HERV-K_copy_chr3-5	3q27.2	chr3:185563474-185563838	Yes
HERV-K_copy_chr5-1	5p13.3	chr5:30487571-30487935	
HERV-K_copy_chr5-2	5q33.3	chr5:156658632-156658996	Yes
HERV-K_copy_chr6	6q14.1	chr6:77717863-77718227	Yes
HERV-K_copy_chr7-1	7p22.1a	chr7:4583352-4583716	Yes
HERV-K_copy_chr7-2	7p22.1b	chr7:4591856-4592220	Yes
HERV-K_copy_chr8-1	8p23.1a	chr8:7498801-7499165	Yes
HERV-K_copy_chr8-2	8q24.3a	chr8:139460832-139461196	Yes
HERV-K_copy_chr10	10p14	chr10:6825105-6825469	
HERV-K_copy_chr11-1	11q22.1	chr11:101703237-101703601	Yes
HERV-K_copy_chr11-2	11q23.3	chr11:118721940-118722304	
HERV-K_copy_chr12	12q14.1	chr12:58328385-58328749	Yes
HERV-K_copy_chr16-1	*	chr16:34412975-34413339	
HERV-K_copy_chr16-2	16p11.2	chr16:34998490-34998854	Unknown
HERV-K_copy_chr19-1	19p12c	chr19:22580468-22580832	
HERV-K_copy_chr19-2	19q11	chr19:27638543-27638907	Yes
HERV-K_copy_chr19-3	19q13.12a	chr19:35572396-35572760	
HERV-K_copy_chr22	22q11.21	chr22:18946561-18946925	Yes

The first column lists the abbreviated code for different copies throughout this study. Codes suggested in Subramian et al., 2011 [20] are listed in the second column (* indicates not listed), and are based on chromosome band location in the human genome. Genomic localization (GRch 38.92 version of the *Homo sapiens* genome) is represented in the third column and uniqueness to the human genome is also indicated (again based on Subramian et al., 2011 [20]).

Table S5. List of set of primers used for the qPCR expression analysis.

Target	Sequence (5'-3')	1 mM $MgCl_2$ addition
HERVK *ENV* (Li et al., 2015)	For: CTGAGGCAATTGCAGGAGTT<br>Rev: GCTGTCTCTTCGGAGCTGTT	No
HERVK *POL* (Li et al., 2015)	For: TCACATGGAAACAGGCAAAA<br>Rev: AGGTACATGCGTGACATCCA	No
GRANZYME B	For: GACAGCTGCTCACTGTTGGG<br>Rev: ATAGGCTGGATGGGGGATGG	No
hGAPDH	For: ATCAGCAATGCCTCCTGCAC<br>Rev: TGGCATGGACTGTGGTCATG	No

The first column shows the gene target, the second column indicates the primer sequences (5'-3'; For= forward; Rev= reverse) and the third column points out whether reactions were supplemented with 1 mM $MgCl_2$ or not.
